# Rehabilitation Following Surgery for Reconstruction of a Foot Defect

**DOI:** 10.4137/ccrep.s726

**Published:** 2008-04-15

**Authors:** Susan Faber West, Peter E. Pidcoe

**Affiliations:** 1 Department of Physical Therapy Outpatient Team, Medical College of Virginia Hospitals, Richmond, Virginia, U.S.A.; 2 Department of Physical Therapy, Virginia Commonwealth University—Medical College of Virginia, Richmond, VA 23298.

**Keywords:** arterio-venous malformation, musculocutaneous flap, scar management, pressure garments, remolding, wound care

## Abstract

**Background:**

This report illustrates the use of pressure for scar management to aid in foot re-shaping following a surgical intervention to repair an arterio-venous (AV) malformation.

**Methods:**

This report describes the rehabilitation of a 13-year-old girl after surgical reconstruction of a defect in her left foot following the removal of an AV malformation. Early surgical attempts to repair the problem resulted in complications that required the amputation of toes 2, 3, and 4, and the use of a split thickness skin graft to cover the plantar surface of the medial longitudinal arch on the left foot. Following surgery, the patient had an antalgic gait pattern with decreased weight bearing on the left. The graft obliterated the left medial longitudinal arch and the patient would only weight bear on the heel. The patient had decreased metatarsal joint mobility on the affected side and no movement in the remaining toes. Left talocrural joint active range-of-motion (AROM) was within normal limits and gross ankle muscle force production was assessed to have a grade of 3/5.

**Results:**

Treatment included reshaping the left foot using a pressure garment and orthotic, followed by interventions to address range-of-motion and muscle force production deficiencies. All treatment objectives were achieved and all patient goals were achieved.

**Conclusions:**

Pressure was effective in re-shaping the foot to promote normal gait mechanics.

## Introduction

The purpose of this report is to illustrate the use of pressure for scar management to aid in re-shaping a foot following a surgical intervention to repair an arterio-venous (AV) malformation. An AV malformation, or fistula, can be defined as a “connection, other than the capillary bed, between the arterial and venous system”. The definition encompasses a vast array of conditions, including some occurring in the normal development of circulation, congenital malformations, acquired lesions, and iatrogenic shunts (Lyerley and Sabiston, 1997). This case report describes the rehabilitation of a 13-year-old girl after surgical reconstruction following the repair of an AV malformation. The reconstruction included the use of a split-thickness skin graft on the plantar surface of the patients left foot. The graft and surgical scar obliterated the left medial longitudinal arch and contributed to an antalgic gait pattern with decreased weight bearing on the left. The patient was unable to wear normal shoes. A pressure garment and orthotic were used in the treatment of this scar in an effort to remodel the tissue. In addition, the patient was treated for proximal muscle weakness, deconditioning, and motor coordination deficits.

In recent literature, there are many references to the use of microvascular muscle flaps for the reconstruction and repair of foot defects (Weinzweig and Davies, 1998; Cooper et al. 1999). Although graft donor sites differ, the reconstruction process is similar. The intent of the surgery was to repair the defect to create a suitable weight-bearing surface that would help restore normal foot mechanics. Gait analysis of patients who have received these grafts has confirmed the weight-bearing capabilities of free muscle flaps with skin grafts (May et al. 1985). Long-term follow-ups of patients with these types of grafts, however, have revealed a frequent need for secondary debulking procedures to improve the function and weight-bearing capabilities of the individual (Goldberg et al. 1993). This is often due to hypertrophic scarring around the graft and is more prevalent in the pediatric population as compared to adults (Harris et al. 1994).

A historical perspective presented by Linares (1993), Larson et al. reports that the first medical reference on the use of pressure for scar management was from the works of Ambroise Pare in the sixteenth century. Subsequent references include Verneuil in 1860, Panas in 1863, and Dr. Unna with the use of circular strips of adhesive plaster in 1881. Nason reported in 1962 that overproduction of scar tissue is inhibited by ischemia resulting from pressure. If fact, it has been proposed that the pressure influences the realignment of the collagen bundles in the forming scar (Johnson, 1984). The pressure garment (or pressure wrap) is typically designed to produce skin pressure between 25 and 32 mmHg, exceeding the average capillary pressure of 20 mmHg (Wienert, 1999). These pressures may average as low as 5 to 15 mmHg, but have been shown to still advance a positive clinical response (Cheng et al. 1984). The use of pressure for scar management became standard treatment for burns after the studies conducted in the Shriners Burns Institute, Galveston Unit in 1968 (Linares et al. 1993). The use of continuous pressure in the management of burn scars is historically well documented and well accepted (Kisher et al. 1975; Torring, 1984; Kealey and Jensen, 1988; Ward, 1991).

In 1978, it was reported that the use of compression garments was effective in controlling scarring due to burns in children (Garcia-Velasco et al. 1978). Cheng et al. agreed that pressure therapy was effective in preventing and correcting hypertrophic scarring after burn injury. They added that pressure should be standardized over the surface of the garment scar interface and recommended the use of pressure padding (Cheng et al. 1984). This approach was applied to the patient described in this case report. With this patient, the musculocutaneous flap used in reconstructive surgery obliterated the medial longitudinal arch of her left foot. This resulted in a convex plantar surface on that foot. The foot shape inhibited normal foot mechanics during weight-bearing activities. One of the physical therapy treatment goals was to reshape the immature scar in an effort to enhance function. A secondary goal was to create a foot shape that would make is possible for the patient to wear normal footwear. Pressure padding was provided by an of-the-shelf arch support.

## Purpose

The purpose of this case report is to describe the multifaceted rehabilitation of a 13-year-old girl following reconstruction of a defect in her left foot after the removal of an AV malformation.

## History

Information was obtained from chart notes and surgical reports from the department of plastic surgery, Medical College of Virginia Hospitals, Richmond, Virginia.

### Pre-surgical evaluation

The patient was a 13-year-old female who was brought to the United States by her uncle in the summer of 1997 to have an ulcer on her left foot evaluated and treated. Past medical history revealed that the patient recalled that she had had some sort of a lesion on the left foot since she was two years old. The lesion was described as being mostly on the plantar surface of the foot. When the patient was approximately six years old, treatment was provide in Turkey that resulted in an ulcer that had never healed as well as a necrotic area on the plantar surface of the foot. This treatment probably included some kind of freezing or coagulation of the superficial part of the arterio-venous malformation.

**Figure 1:**
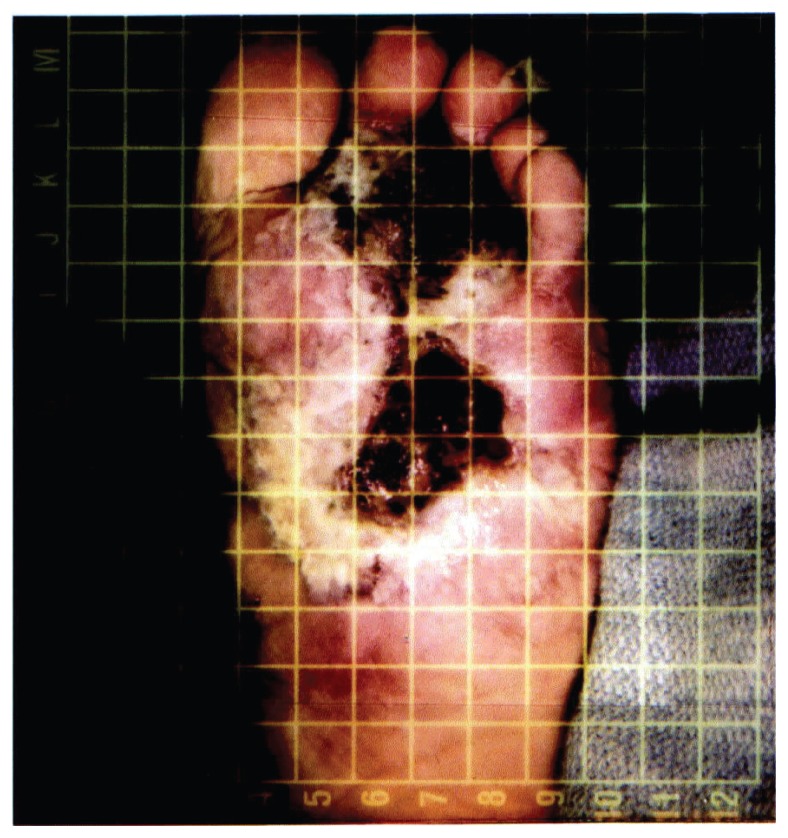
Plantar surface of the foot at time of pre-surgical evaluation.

The patient was seen by the plastic surgery service at Medical College of Virginia on August 6, 1997. During hospitalization, the patient was diagnosed with a very high outflow AV malformation with rapid venous filling (as measured via angiography). Embolization was attempted using a branch of the posterior tibial artery, but the main feeding branches were too small to be embolized successfully. Upon physical examination of the left foot by the plastic surgeon the metatarsophalangeal joints were found to be in flexion. There was significant edema of the plantar and dorsal aspects of the foot. On the plantar surface of the left foot, there was an ulcer approximately three by five centimeters in the midfoot area and an area of necrotic tissue approximately five by five centimeters distal to that (Fig. 1).

There was evidence of venous congestion and discoloration of the skin involving toes two, three and four. There was also a venous thrill over the anterior tibial and posterior tibial areas. Sensation was decreased but the patient reported pain with light touch over the discolored area. The surgeon hypothesized that there may have been some shortening of the femur and tibia and decreased muscle mass in the entire left leg. The treatment options stated in the surgical consultation were to: (1) do nothing, (2) perform a mid-tarsal amputation, or (3) remove the affected tissue and replace with a muscle flap and split thickness skin graft.

### Surgery

Five weeks after the initial evaluation, option 3 was selected (removal of the affected tissue and surgical reconstruction using a muscle flap and split-thickness skin graft). The AV malformation including toes two, three, and four was excised. A free flap from the rectus abdominis muscle was placed in the void and covered with split thickness skin graft. The rectus abdominis muscle flap failed and was replaced by latissimus dorsi muscle flap four weeks later. The second flap was successful.

### Physical therapy

#### Week 1

**Figure 2:**
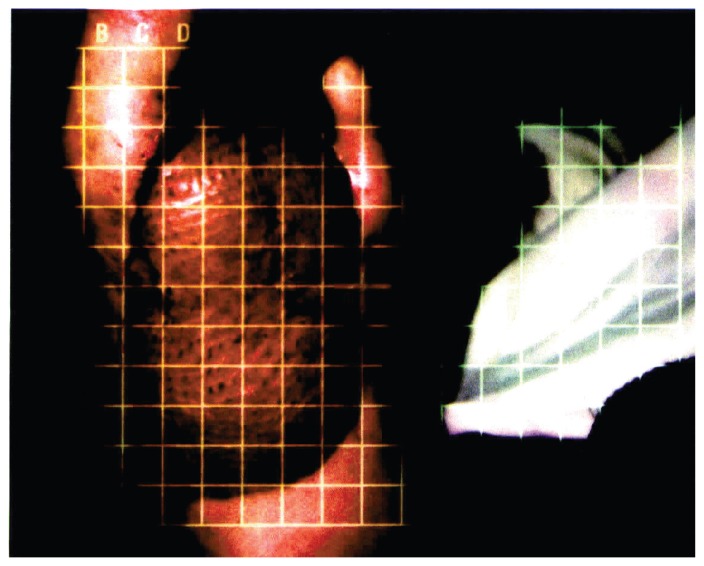
Immature flap and graft over plantar surface of left foot at the time of initial physical therapy evaluation.

The patient was referred to physical therapy in December 1997. This was eight weeks status-post the second reconstructive surgical procedure. Initial findings included: The patient ambulated with severe left antalgic gait pattern. She had normal weight bearing on the right foot, but on her left side was only weight bearing on the heel. The graft used in the reconstruction was immature and rounded over the plantar surface of the foot (Fig. 2). It completely obliterated the medial longitudinal arch of the left foot and was firm to palpation. The patient normally wore size eight shoes, but required a size nine shoe to accommodate the graft. The patient had decreased joint mobility at the tarso-metatarsal (TMT) joints as compared to the uninvolved side and no movement in the remaining toes. The 1st and 5th metatarsophalangeal (MTP) joints of the involved foot were fixed in 0° for flexion and extension. Left talocrural joint active range of motion (AROM) was DF = 20° and PF = 45°. Gross ankle muscle force production was assessed and found to have a grade of 3/5. The ankle range of motion finding was unexpected since the patient had had a severe gait deviation for most of her life. For this same reason, the Fair strength rating of the ankle muscles was not surprising.

##### Treatment objectives included

1. Control and reduce postoperative edema of the left lower extremity.
2. Increase range-of-motion (ROM) at the 1st MTP joint on the left foot from 0° to at least 20° of extension to promote normal foot mechanics during ambulation. Mean ROM values for the 1st MTP joint are 70° extension and 45° flexion (American Academy of Orthopedic Surgeons: Joint Motion: Method of Measuring and Recording, 1965). However, 20° of extension and 20° of flexion are considered to be within normal limits (Donatelli, 1990). This ROM is important due to the mechanical relationship between 1st MTP extension and the formation of the medial longitudinal arch during the toe-off phase of gait (Bojsen-Moller and Lamoreux, 1979). The formation of the arch allows the foot to become a more rigid structure, facilitating the efficient transfer of forces to the weight-bearing surface.
3. Increase the force production capability of muscles of the left ankle from a grade of 3/5 to 4/5 to promote normal foot mechanics during ambulation and the performance of ADLs.
4. Reshape the grafted area to create a more normal foot shape. As previously noted, the graft had obliterated the medial longitudinal arch of the left foot. In effect, the graft created a foot that was similar in its mechanical operation to a rocker-bottom shoe. Reshaping the grafted area to regain the plantar arch should promote more normal foot mechanics during ambulation.

**Figure 3:**
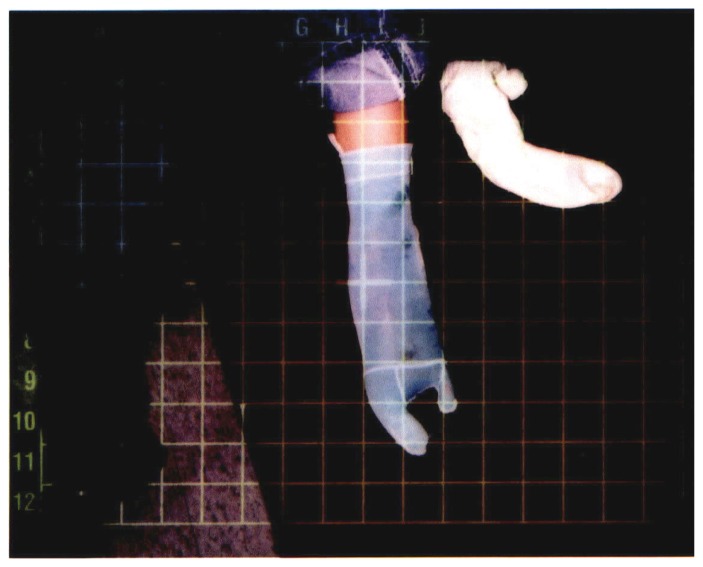
Custom made pressure garment for left foot and ankle.

The patient was to be seen twice a week for an expected period of 12 weeks. Of highest priority was the control of postoperative edema of the left lower extremity. The patient was referred to occupational therapy for a custom measured pressure garment for the foot and ankle (Fig. 3). Circumferential measurements are reported and discussed later in this case report with comparison to measurements taken in week 11 of physical therapy. The patient was also referred to an orthotist for custom-made arch supports for both feet. The purpose of arch supports was to facilitate the reshaping of the arch of the left foot by supplying pressure to the area of the medial longitudinal arch while weight bearing.

The first week of physical therapy included instructions in a home exercise program. This program included passive mobilizations for the metatarsal joints and strengthening exercises for the left ankle, using red Theraband (resistive weight 3.3 lbs). On the second physical therapy visit (5 days after initial evaluation), palpation of the left foot revealed that the plantar surface of the grafted area appeared softer. There was no evidence of that the patient could actively move the fifth toe on her left foot, but she was able to move the great toe through 20° of extension and 10° of flexion actively. The patient was able to ambulate slowly using a standard cane on the right. The patient presented with no gait deviations except for a slight right gluteus medius limp. Observations of the plastic surgeon noting possible left LE shortening were found to be functionally insignificant.

#### Week 2

At the beginning of the second week of therapy the bilateral arch supports were obtained. Therapy continued and now included gait components on level surfaces and stairs. It was discovered that the patient had difficulty with eccentric control of the left quadriceps muscles when descending stairs. It was felt that this control deficit was due, in part, to a decreased muscle force production capability of the knee extensors. In an effort to address this problem (and the gluteus medius limp noted earlier), quadriceps strengthening exercises and hip abductors exercises were introduced into the home exercise program. These exercises included straight leg raises in the frontal and sagittal planes.

Patient deconditioning was noted at this point in the treatment program as a result of the increased endurance demands of ambulation. This was evidenced by the fact that the patient could run only 200 feet before becoming short of breath. Physical therapy treatment frequency was increased to three times per week to allow an increased focus on endurance protocols.

#### Week 3

For the next 6 weeks the patient was followed three times a week for left lower extremity strengthening, gait training, and endurance training.

#### Week 6

Circumferential measurements of both lower extremities were taken after approximately 6 weeks of therapy to quantify observed asymmetries. The results are tabled below (Table 1):

It can be seen from these data, that there is a measurable difference in the circumference of the lower extremities. The left (or involved) leg is smaller than the right, suggesting a muscle imbalance since muscle force generation capacity is directly related to cross-sectional area (Roy and Edgerton, 1992). At this stage of physical therapy treatment, the patient was directed to begin exercises for general strengthening the left lower extremity musculature including increased attention to the ankle dorsiflexor muscles previously identified as deficient. The patient’s main goals were to prepare to take dance classes and to participate in general health and physical education classes by the time she returned home. The patient continued to be followed two times weekly for cardiovascular endurance training and lower extremity strengthening.

#### Week 11

The final physical therapy visit was during the 11th week of treatment. The patient reported that she was very happy with the results of her surgery and therapy. Circumferential measurements of the left lower extremity were repeated. These data are presented in the table below (Table 2) along with comparative data from week 6 of treatment. The results indicate a general muscle hypertrophy in the left lower extremity from week 6 to week 11.

**Figure 4:**
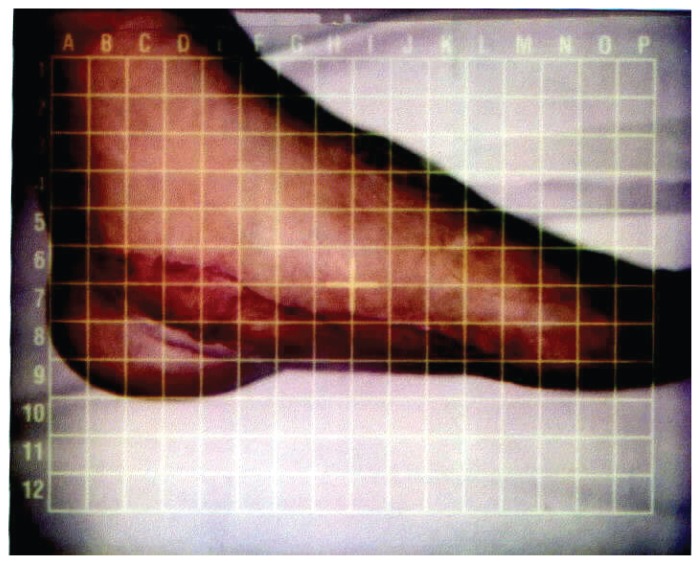
Plantar arch of left foot after remolding with garment and arch support.

Pressure garment measurements were repeated at this time and a new pressure garment was issued. The measurement results are provided in Table 3 and reveal a general decrease in the size of the left foot in the area of the graft site. The size of the graft site on the left foot had been effectively remolded to create a more functional foot shape. At the conclusion of therapy, the patient was able to wear size 8 shoes on both feet (Fig. 4). ROM measures for 1st MTP flexion/extension where 10° and 20° respectively. Manual muscle testing revealed left PF = 4/5 and DF = 3/5. The patient did not improve in DF muscle force production, but this did not impede normal gait mechanics and functional ambulation. All treatment goals were achieved and the patient was discharged.

## Discussion

The rehabilitation of this patient can be described as multifaceted. The three months the patient spent in physical therapy included treatments to reshape her foot, improve the ROM of the foot and ankle, gait normalization to create a more functional pattern, LE muscle strengthening, and treatment for general deconditioning. There was no literature or protocol available to provide direct support in the rehabilitation of patients with similar problems. It was also found that there is a lack of reference material related to the treatment of deconditioned children. The physical therapy treatment protocol centered on the most obvious deficits first and was modified to accommodate other clinical problems as they became apparent.

The graft used on this patient was a free latissimus dorsi plus split-thickness skin graft. Its use for this type of defect is supported in the literature. (Santanelli et al. 2002; Baudet et al. 1976). One benefit is a reduction in the thickness of the repair. Fasciocutaneous flaps are also an option, but regardless of the type of free flap used, postoperative morbidity is best minimized by good preoperative planning and attending to the geometry of the anatomical defect when selecting and placing the graph (Wyble et al. 1990). In this case report, additional reduction and contouring of the repair was achieved over the course of 11 weeks of rehabilitation with the use of a fitted pressure garment.

One limitation of this case study is the lack of objective sensory data on and around the graph site pre and post rehabilitation. The return of protective sensation is important in the prevention of foot ulcers (Kuran et al. 2000). This return is expected within 12 to 24 months post surgery, with most graph sites beginning to show sensory recovery in four to five weeks (Gideroglu et al. 2005). This occurs through two pathways: in-growth from the surrounding nerves in the periphery and re-growth from surgically create sensory neural pathways (Kimata et al. 1999). Protective sensation thresholds can be measured in a several ways. Semmes-Weinstein monofilament detection between 5.07 and 5.46 has been suggested as an acceptable cutoff to decrease the potential for ulceration of the graph site (Gideroglu et al. 2005; Jeng et al. 2000). This is a widely available clinical tool. Vibration and somatosensory evoked potentials are also used. Although the therapist would have no control of the rate or magnitude of sensory return, monitoring sensation would provide valuable information that should be part of the patient education process.

The progression of therapy in this case illustrates the need to constantly re-evaluate the needs and progress of the patient. Following the initial evaluation, subsequent visits to physical therapy revealed new problem elements that were initially masked by other problems. The therapy was constantly being revised to address these surfacing problems. Since AV malformation was present at birth, the patient never developed what would be classically characterized as a normal gait pattern. Therapy involved a large component of neuromuscular reeducation. In retrospect, the rehabilitation process concentrated primarily on general gross lower extremity function and progressive endurance training as opposed to soft tissue mobilization and facilitation of fine movements of the foot that were initially anticipated.

## Figures and Tables

**Table 1:** Week 6 lower extremity circumferential measurements.

Measurement location	Left LE circumference (cm)		Right LE circumference (cm)
20 cm superior to inferior pole of patella	46.00	<	48.50
Inferior pole of patella	32.00	<	34.00
5 cm below inferior pole of patella	31.25	<	35.50
10 cm below inferior pole of patella	29.00	<	35.35
15 cm below inferior pole of patella	26.50	<	32.00
20 cm below inferior pole of patella	24.00	>	20.00

**Table 2:** Comparison of lower extremity circumferential measurements for week 6 and 11.

Measurement location	Week 6 Left LE circumference (cm)		Week 11 Left LE circumference (cm)
20 cm superior to inferior pole of patella	46.00	>	44.50
Inferior pole of patella	32.00	=	32.00
5 cm below inferior pole of patella	31.25	<	32.50
10 cm below inferior pole of patella	29.00	<	30.50
15 cm below inferior pole of patella	26.50	<	27.50
20 cm below inferior pole of patella	24.00	=	24.00

**Table 3:** Circumferential measurements used for the fabrication of a pressure garment for the left foot and ankle. Data shows measurements from week 1 and week 11 of treatment. These measurements were originally taken in inches, but have been converted to cm for reporting consistency. The diagram to the right identifies the location of the measurements. 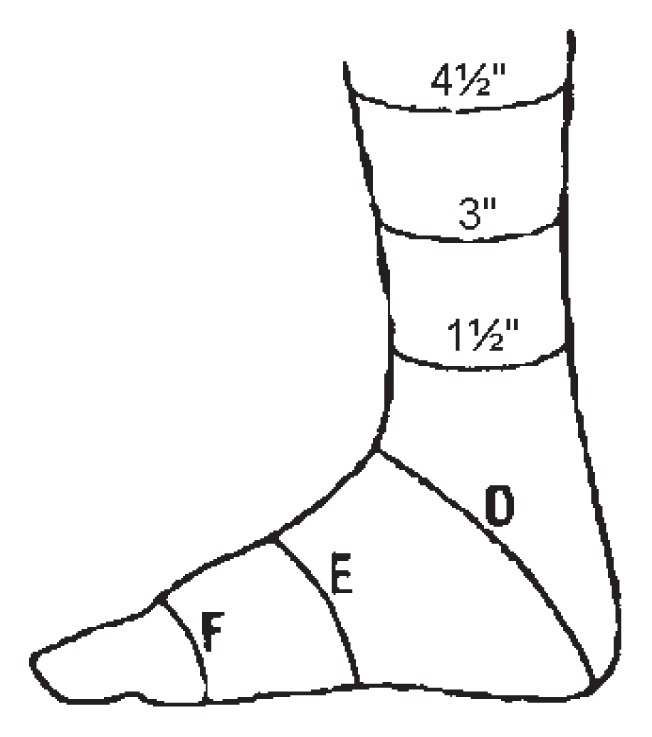

Measurement location	Week 1 Left LE circumference (cm)		Week 11 Left LE circumference (cm)
4½″ proximal to lateral malleolus	20.96	<	21.27
3″ proximal to lateral malleolus	18.73	>	18.57
1½″ proximal to lateral malleolus	18.42	<	18.73
D	31.12	>	30.16
E	23.18	>	22.70
F	20.00	>	19.84
